# A novel necroptosis-related lncRNA signature predicts the prognosis and immune microenvironment of hepatocellular carcinoma

**DOI:** 10.3389/fgene.2022.985191

**Published:** 2022-10-04

**Authors:** Jianguo Wang, Bingbing Shen, Xinyuan Liu, Jianxin Jiang

**Affiliations:** ^1^ Department of Hepatobiliary Surgery, Renmin Hospital of Wuhan University, Wuhan, Hubei, China; ^2^ Department of Hepatic-Biliary-Pancreatic Surgery, The Affiliated Hospital of Guizhou Medical University, Guiyang, Guizhou, China

**Keywords:** hepatocellular carcinoma, lncRNA, bioinformatics, necroptosis, risk model, immune infiltration

## Abstract

Hepatocellular carcinoma (HCC) is one of the malignant tumors with high mortality and a worse prognosis globally. Necroptosis is a programmed death mediated by receptor-interacting Protein 1 (*RIP1*), receptor-interacting Protein 1 (*RIP3*), and Mixed Lineage Kinase Domain-Like (*MLKL*). Our study aimed to create a new Necroptosis-related *lncRNAs* (*NRlncRNAs*) risk model that can predict survival and tumor immunity in HCC patients. The RNA expression and clinical data originated from the TCGA database. Pearson correlation analysis was applied to identify the *NRlncRNAs*. The LASSO-Cox regression analysis was employed to build the risk model. Next, the ROC curve and the area under the Kaplan-Meier curve were utilized to evaluate the accuracy of the risk model. In addition, based on the two groups of risk model, we performed the following analysis: clinical correlation, differential expression, PCA, TMB, GSEA analysis, immune cells infiltration, and clinical drug prediction analysis. Plus, qRT-PCR was applied to test the expression of genes in the risk model. Finally, a prognosis model covering six necroptosis-related *lncRNAs* was constructed to predict the survival of HCC patients. The ROC curve results showed that the risk model possesses better accuracy. The 1, 3, and 5-years AUC values were 0.746, 0.712, and 0.670, respectively. Of course, we also observed that significant differences exist in the following analysis, such as functional signaling pathways, immunological state, mutation profiles, and medication sensitivity between high-risk and low-risk groups of HCC patients. The result of qRT-PCR confirmed that three NRlncRNAs were more highly expressed in HCC cell lines than in the normal cell line. In conclusion, based on the bioinformatics analysis, we constructed an *NRlncRNAs* associated risk model, which predicts the prognosis of HCC patients. Although our study has some limitations, it may greatly contribute to the treatment of HCC and medical progression.

## Introduction

Hepatocellular carcinoma (HCC) is one of the most common malignancies and the leading cause of worldwide cancer-related death. According to the statistics on liver cancer in the GLOBOCAN2020 edition, there were about 905,677 new cases and 830,180 deaths worldwide, making it sixth-largest cancer and the third leading cause of tumor-related death in the world (Sung et al., 2021). There is also a significant geographic difference in the incidence of HCC, with most cases occurring in Asia. The main known risk factors associated with hepatocellular carcinoma are viral (chronic hepatitis B and C), metabolic (diabetes mellitus and non-alcoholic fatty liver disease or NAFLD), toxic (alcohol and aflatoxins), and immune system-related diseases (Chakraborty and Sarkar, 2022). At present, hepatectomy and liver transplantation have become the primary treatment methods for patients with early hepatocellular carcinoma, while hepatic arterial chemoembolization and radioembolization are often used for intermediate-stage patients, which can significantly prolong the survival of patients (Cillo et al., 2014; Xue et al., 2015; Marrero et al., 2018). There has been considerable progress in treating liver cancer over the past few decades, but the prognosis is still poor for HCC patients. Therefore, it is crucial to explore nocel prognosis biomarkers and therapies to improve the prognosis and treatment of HCC patients.

The long noncoding RNA (*lncRNA*) is a transcriptional RNA with over 200 nucleotides but does not translate into protein (Wang et al., 2018). Previous studies have shown that unnatural *lncRNA* expression frequently occurs in various cancers and numerous biological processes such as tumor proliferation, invasion, and development (Huarte, 2015). *LncRNA* has been proved to have an abnormal expression in HCC and participate in cancer phenotypes, such as continuous proliferation, avoidance of apoptosis, acceleration of blood vessel formation, and acquisition of invasion ability (Huang et al., 2020). It means *lncRNAs* can be used as cancer progression markers and potential therapeutic targets.

A controlled form of necrosis, necroptosis is a necrotic cell death unmediated by caspases, mediated primarily by receptor-interacting Protein 1 (*RIP1*), receptor-interacting Protein 1 (*RIP3*), and Mixed Lineage Kinase Domain-Like (*MLKL*) (Gong et al., 2019). An early sign of necroptosis is the loss of integrity of the plasma membrane, leakage of intracellular contents, and organelle swelling (Krysko et al., 2017). Recent studies have found that necroptosis plays an essential role in tumorigenesis, tumor metastasis, and tumor immunity (Jiao et al., 2018). Activating the CXCL5-CXCR2 axis at the pancreatic cancer invasion front due to necroptosis can increase cancer cell migration and invasion (Ando et al., 2020). By promoting *RIPK3-MLKL*-mediated necroptosis, *RIPK3* may be able to limit the occurrence of myeloid leukemia and the differentiation of leukemia initiation cells (Höckendorf et al., 2016). There are still questions about what mechanism necrotic disease plays in tumor regulation, and no research has not been done on the role of Necroptosis-related l*ncRNAs* in HCC.

We developed a new predictive feature in this study to predict the prognosis of HCC based on Necroptosis-related *lncRNAs* (*NRlncRNAs*). In addition to validating its clinical value, we also confirmed the use of the score as a predictor of immunotherapy, which could provide clinicians with guidance.

The detailed flow diagram of our study is shown in Supplementary Figure S1.

## Materials and methods

### Data collection

RNA sequencing (RNA-seq) data and clinical features were retrieved from the TCGA database (https://portal.gdc.cancer.gov/). We also downloaded Copy Number Variation (CNV) data of HCC patients from the TCGA database. According to previously reported literature, 67 necroptosis genes have been identified (Zhao et al., 2021).

### Identification and expression analysis of nrcroptosis-related lncRNAs

The transcriptome data were separated into lncRNA and mRNA using the Strawberry Perl program. Pearson correlation analysis and co-expression analysis were applied to identify Necroptosis-related *lncRNAs* (*NRlncRNAs*)l with co-expression correlation coefficient > 0.4 and *p*-value < 0.001. Next, the “Limma” R package was used to conduct the differential expression analysis of *NRlncRNAs*, with |logFC| > 1 and a corrected *p* < 0.05. The “dplyr,” “ggalluvial,” and “ggplot2” R packages were employed to draw the Sankey diagram. The Sankey diagram showed the correlation between necroptosis-related genes and necroptosis-related *lncRNAs*. The “igraph” R package was used to display the *lncRNA-mRNA* network.

### Establishment and validation of a model for assessing prognostic risk

Initially, prognostic-associated *lncRNAs* were identified by using univariate Cox (uni-Cox) regression with *p*-value ≤ 0.05. Subsequently, we conducted the Lasso regression 1,000 times with 10-fold cross-validation and *p*-value ≤ 0.05. Furthermore, we performed multivariate (multi-Cox) proportional hazards regression and risk model construction using the necroptosis-related *lncRNAs* screened by the LASSO method. The following formula was used to determine the risk score: Risk score = Σ coefficient of (*NRlncRNAi*) * expression of (*NRlncRNAi*). Based on the median risk score, HCC samples were divided the HCC patients into low-risk and high-risk groups. The association between clinical characteristics and risk group was validated using the chi-square test. We conducted univariate Cox (uni-Cox) and multivariate Cox (multi-Cox) regression analyses to assess whether the risk score and clinical characteristics were independent prognosis factors. After that, the model precision was evaluated using receiver operating characteristic (ROC) curves and a concordance index (C-index). The “survival,” “caret,” “glmnet,” “rms,” “survminer,” and “timeROC” R packages were used to conduct the studies.

### Nomogram and calibration

Using the “rms” R package, we created a nomogram for the 1-, 3-, and 5-years OS of HCC patients by combining the risk score with clinical characteristics such as age, gender, stage, and tumor stage. Correction curves based on the Hosmer- Lemeshow test were used to show the consistency between the actual outcome and the model prediction outcome.

### Gene set enrichment analyses

GSEA software 4.2.3 (https://www.gsea-msigdb.org/gsea/index.jsp) was applied to carry out GSEA analysis and identify significantly enriched pathways. The statistical significance criteria were set at *p* < 0.05 and FDR<0.25, respectively. This process used the “plyr,” “gridExtra,” “grid,” and “ggplot2” R packages.

### Prognostic signature estimation of the tumor immune microenvironment

To determine the association between this signature and TIME (Tumor Immune Microenvironment), we used seven algorithms to predict infiltration values for TCGA-LIHC dataset samples, such as XCELL, TIMER, QUANTISEQ, MCPCOUNTER, EPIC, CIBERSORT-ABS, and CIBERSORT. Then, we analyzed differences in the expression of immune and stromal cells between patients in high and low-risk groups. Using the “limma” and “estimate” R packages, each patient’s StromalScore, ImmuneScore, and ESTIMATEScore (StromalScore + ImmuneScore) were calculated. The Wilcoxon-signed-rank test was used to compare the score differences between the high-risk and low-risk groups, and *p* < 0.05 was considered significant. Using the “GSVA” R package, single-sample GSEA (ssGSEA) was used to score HCC-infiltrating immune cells and quantify their relative content. The scores of immune cells and pathways in different groups are displayed on multi-box plots. Finally, the “ggpubr” R package was applied to paint the correlation graph of immune checkpoints and risk groups.

### Tumor Immune Dysfunction and Exclusion, N6-methyladenosine (m6A)-related genes, and stem cell-like features

The Wilcoxon signed-rank test was used to examine the expression of m6A-related genes (eight writers, 13 readers, and two erasers) between the low- and high-risk groups. Furthermore, the association between the risk score and tumor stemness was measured using Spearman correlation analysis. Finally, we explored differences in TIDE scores between high-risk and low-risk groups of patients using the Tumor Immune Dysfunction and Exclusion (TIDE) database (http://tide.dfci.harvard.edu/).

### Clusters based on prognostic NRlncRNAs

Based on NRlncRNA expression related to the risk model, the “ConsensusClusterPlus” R package was employed to explore possible Molecular subgroups of responding to immunotherapy, and we obtained the three subtypes. The “survival”, “Rtsne,” “limma,” and “reshape2” were applied to assess the cluster subtypes.

### Tumor mutation burden

The risk score was used to separate the original mutation annotation format (MAF) into two groups. Then, the tumor mutation burden (TMB) score in the high-risk and low-risk groups was determined based on somatic mutation data. The previous study used the “Maftools” R package.

### Prediction of chemotherapy and target agent response

We calculated IC50 values for medications collected from the GDSC website (https://www.cancerrxgene.org/) to anticipate prospective compounds employed for HCC treatment. The package “oncoPredict” investigated the therapeutic effect of medicines in high-risk and low-risk groups.

### Quantitative real-time PCR

Before the qRT-PCR procedures, we extract total RNA with TRIzol reagent (Thermo Fisher Scientific. Inc.). Next, reverse transcription was used to produce cDNA with a Thermo Fisher Scientific reagent Kit. The qRT-PCR was performed on the Quantagene q225 real-time PCR system.

### Statistics analysis

R studio and GraphPad Prism were applied to conduct the statistical analysis in the study. Differences were regarded as statistically significant for *p*-values <0.05.

## Results

### Identification of necroptosis-related lncRNAs in hepatocellular carcinoma patients

**FIGURE 1:**
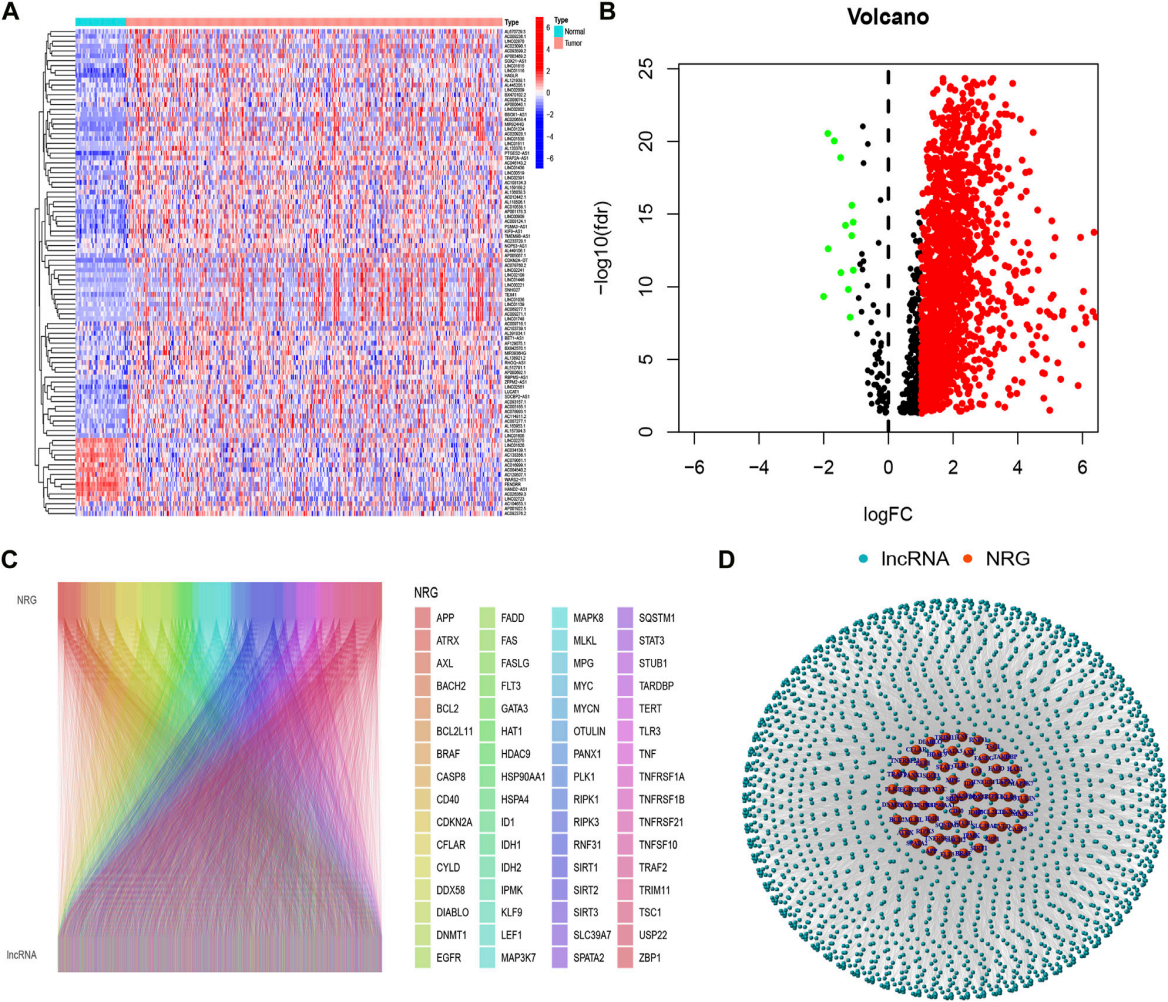
Identification of necroptosis-related *lncRNAs*. **(A)** Heatmap of differentially expressed *NRlncRNAs*; **(B)** volcano diagram of differentially expressed *NRlncRNAs*; **(C)** Sankey diagram of necroptosis-related genes and *lncRNAs*; **(D)** The network necroptosis genes and *lncRNAs*.

Firstly, we acquired the expression data of the TCGA database, which includes 50 normal and 374 tumor samples. Based on the Pearson correlation analysis, we identified 2105 necroptosis-related *lncRNAs*(*NRlncRNAs*), with correlation coefficients > 0.4 and *p* < 0.001. Based on the differential expression analysis of 2105 genes, we obtained 1639 NRlncRNAs (|Log2FC| > 1 and *p* < 0.05) (Figures 1A,B). Subsequently, the Sankey diagram (Figure 1C) and network diagram (Figure 1D) diaplayed the correlation between necroptosis-related genes and NRlncRNAs.

### Construction and verification of prognosis risk model

**FIGURE 2:**
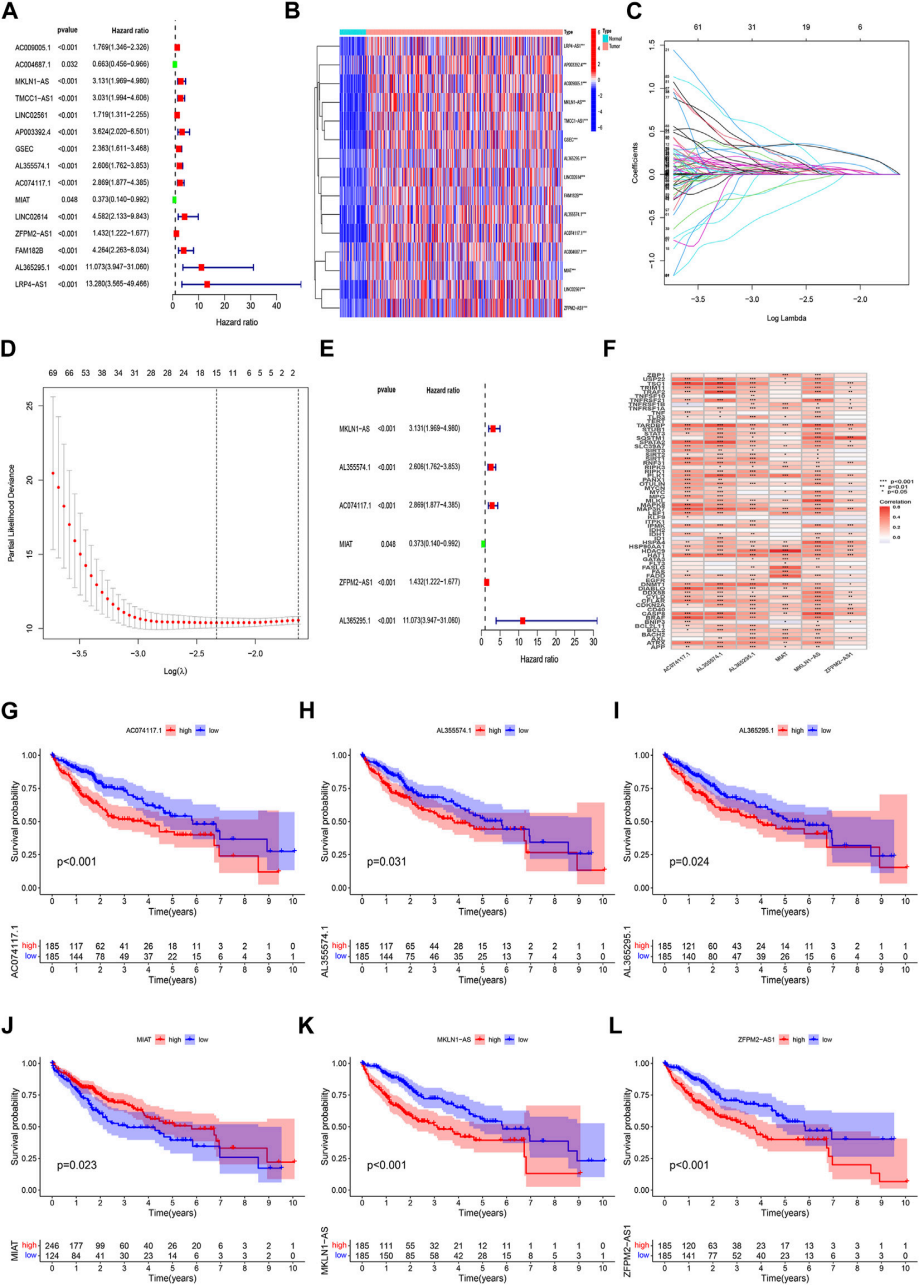
Identification of prognostic *NRlncRNAs*(necroptosis-related *lncRNAs*). **(A)** The prognostic *lncRNAs* obtained by uni-Cox regression analysis; **(B)** The heatmap of 15 prognostic *lncRNAs* expression; **(C–D)** The final *NRlncRNAs* of the prognostic model was confirmed from LASSO regression analysis; **(E)** six *NRlncRNAs* of the risk model; **(F)**Correlations between *NRlncRNAs* in the risk model and necroptosis-related genes **(G)** Kaplan–Meier curve of OS analyzed for AC074117.1. **(H)** Kaplan–Meier curve of OS analyzed for AL355574.1. **(I)** Kaplan–Meier curve of OS analyzed for AL365295.1. **(J)** Kaplan–Meier curve of OS analyzed for MIAT. **(K)** Kaplan–Meier curve of OS analyzed for MKLN1-AS. **(L)** Kaplan–Meier curve of OS analyzed for ZFPM2-AS1.

**FIGURE 3:**
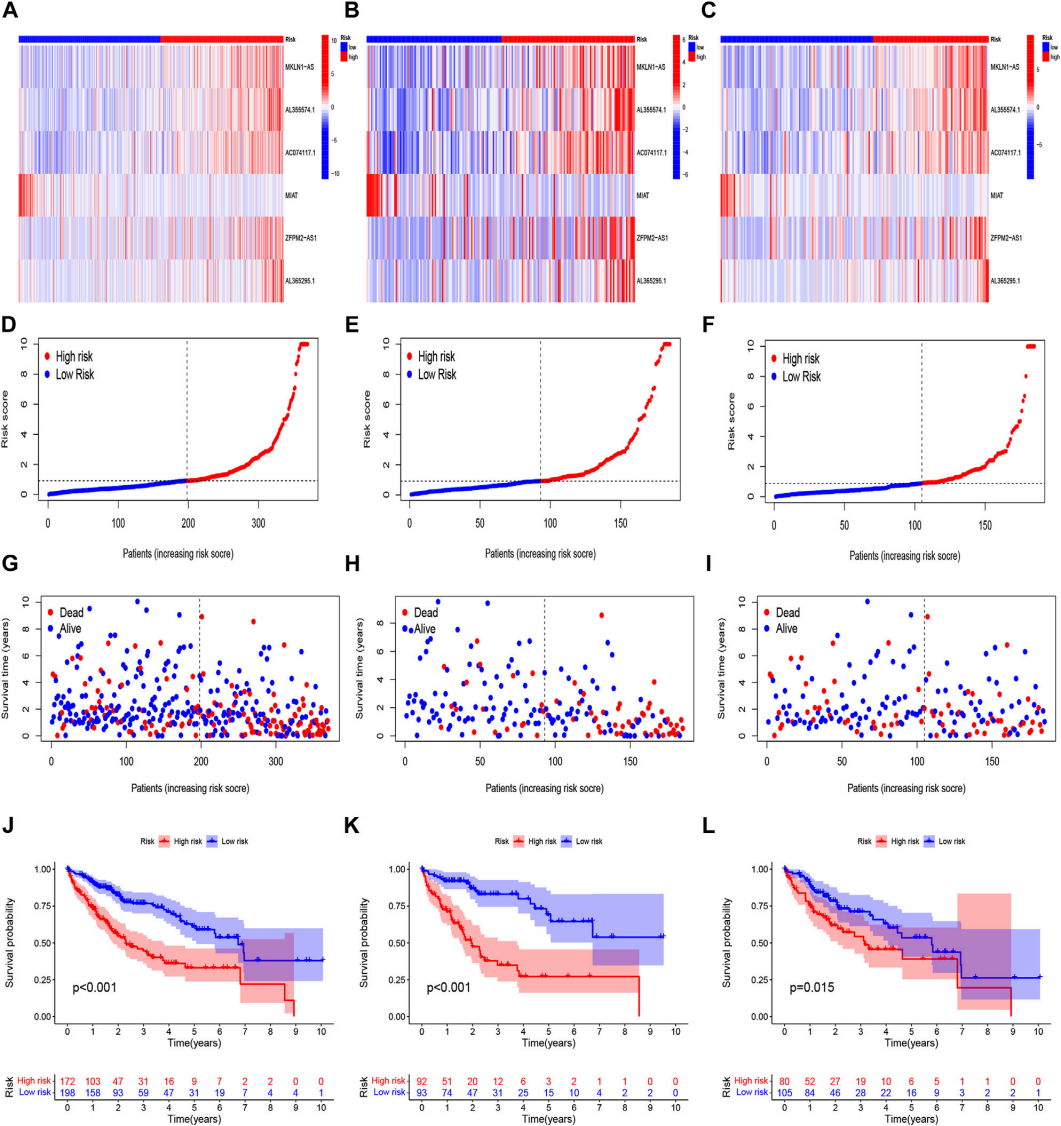
Prognosis evaluation of the risk model. Heatmaps of 6 *lncRNA* expressions **(A–C)**, risk model **(D–F)**, survival time and survival status **(G–I)**, and K-M survival curves of patients with OS **(J–L)** in the entire, train, and test sets, respectively.

**FIGURE 4:**
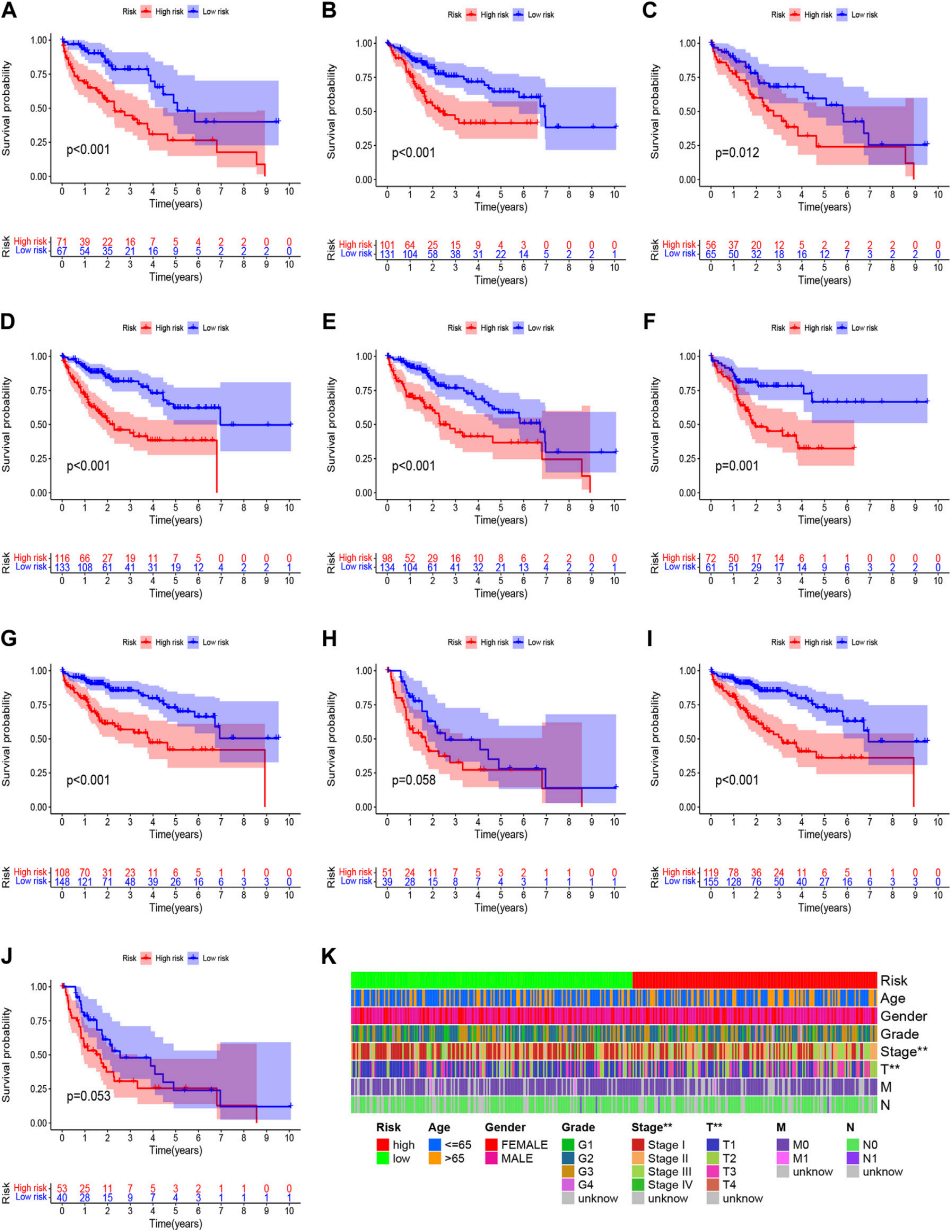
Clinicopathological features analysis in the high- and low-risk score group. **(A)** Patients with age >65; **(B)** Patients with age ≤65; **(C)** Female patients; **(D)** Male patients; **(E)** Patients with Grade G1-2; **(F)** Patients with Grade G3-4; **(G)** Patients with stages I-II; **(H)** Patients with stages III-IV; **(I)** Patients with stages T1-2; **(J)** Patients with stages T3-4; **(K)** Heatmap of clinicopathological features in the high-risk and low-risk group.

**FIGURE 5:**
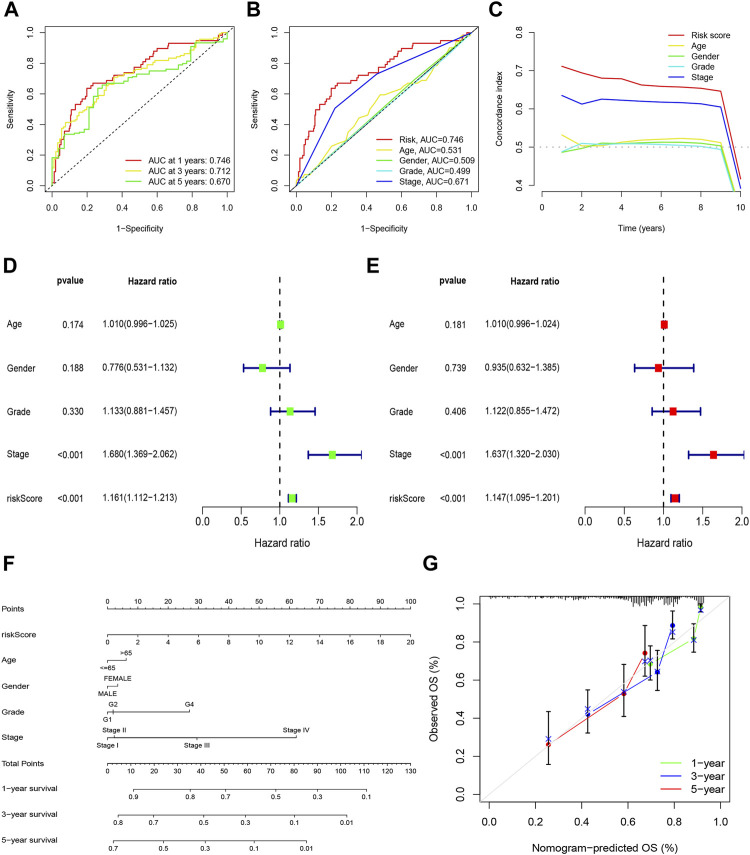
Nomogram and assessment of the risk model. **(A)** The 1-, 3-, and 5-years ROC curves of the entire sets; **(B)** The ROC curves of risk score and clinicopathologic features; **(C)** The C-index curves of risk model; **(D)** Uni-Cox analyses of clinicopathologic factors and risk score with OS; **(E)** Multi-Cox analyses of clinicopathologic factors and risk score with OS; **(F)** Nomogram for predicting OS; **(G)** The calibration curves for 1, 3, and 5 years OS.

Based on univariate Cox regression analysis, we obtained 441 necroptosis-related *lncRNAs* linked with overall survival (OS) (Supplementary Table S1). Subsequently, we acquired 15 *lncRNAs* through LASSO regression analysis, and 6 *lncRNAs* were identified to construct the risk model (Figures 2A–E). Furthermore, the risk score was calculated: Risk score = MKLN1-AS × (0.8260) + AL355574.1 × (0.3672) + AC074117.1 × (0.7261) + MIAT × (-2.0384) + ZFPM2-AS1 × (0.1753) (0.1753) + AL365295.1 × (1.3466). We performed a survival analysis of 6 *lncRNAs*. And results showed that six genes could be regarded as independent prognostic factors of HCC patients (Figures 2G–L). We found that a better prognosis exists in low-risk group compared with high-risk group (Figures 3A–L). Meanwhile, a significant difference exists in the clinical features (Figure 4). Based on the ROC curve, AUC values of 1-, 3-, and 5- years were 0.746, 0.712, and 0.670, respectively (Figure 5A). In addition, the AUC of the risk score was 0.746 for the model (Figure 5B). Plus, the C-index graph (Figure 5C) further showed the survival rate of 1-year is 0.711. Thus, we deem that the risk model possesses a better accuracy. Based on the uni-Cox and multi-Cox regressions analysis, we observed that hazard ratios (HR) of risk score were 1.161 and 1.147, respectively (*p*-value < 0.001) (Figures 5D,E). So, we uncovered that risk score is an independent factor of HCC patients. Finally, a nomogram was designed to estimate the 1-, 3-, and 5-years Overall Survival (OS) rates (Figure 5F). The calibration plots were used to prove if the nomogram had a high level of concordance with the forecast, indicating it was in good agreement with the actual observation (Figure 5G). Therefore, we concluded that the risk model possessed a better accuracy and predicted the OS of HCC patients.

### Risk model is related with tumor immune microenvironment

**FIGURE 6:**
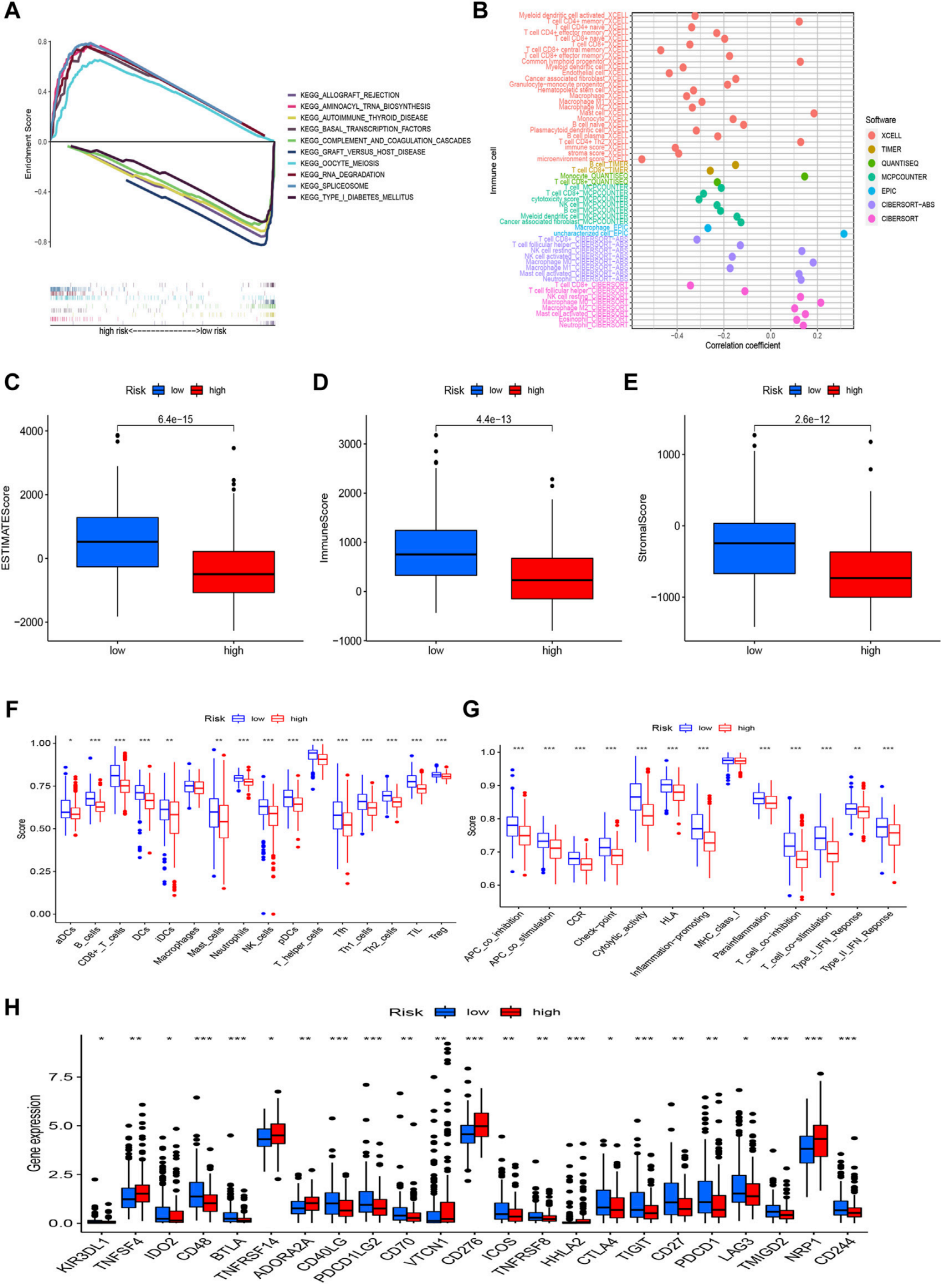
The differences of tumor immune microenvironment between the low-risk and high-risk groups. **(A)** GSEA of the top 10 pathways significantly enriched in the risk groups; **(B)** The immune cell bubble of risk groups; **(C–E)** The boxplots of the comparison of StromalScore, ImmuneScore, and ESTIMATEScore, respectively, between low- and high-risk groups; **(F,G)** The ssGSEA scores of immune cells and immune functions in the risk groups; **(H)** The difference of common immune checkpoint expression in the risk groups. *p < 0.05, **p < 0.01, ***p < 0.001.

We used GSEA to investigate the underlying differences in biological functions based on the various prognoses of patients in high-risk and low-risk groups (Figure 6A). In the low-risk group, we discovered that the complement and coagulation cascades, Autoimmune thyroid disease, Graft-versus-host disease, and Type I diabetes mellitus were highly enriched. The GSEA results also revealed that the high-risk group was considerably enriched in the RNA degradation, Cell cycle, and *mTOR* signaling pathways, Notch signaling pathway, and *Wnt* signaling pathway. These mechanisms could explain why the prognosis for the high-risk group is worse. Of course, we also explored the connection between risk scores and tumor-infiltrating immune cells (Figure 6B). On several platforms, more immune cells are closely associated with the low-risk group. We also discovered that StromalScore, ImmuneScore, and ESTIMATEScore were consistently higher in low-risk than in high-risk patients (Figures 6C–E). In addition, to further investigate the association between risk scores and immune cells and functions, we assessed the enrichment scores of ssGSEA for various immune cell subgroups, related functions, or pathways. According to the results, we discovered that 15 immune cells had higher scores in the low-risk group, covering activated dendritic cells (aDCs), B cells, CD8+_T_cells, DCs, immature dendritic cells (iDCs), mast cells, neutrophils, NK_cells, pDCs, T helper cells, T follicular helper (Tfh) cells, Th1_cells, Th2_cells, tumor-infiltrating lymphocyte (TIL), and T regulatory cells (Tregs) (Figure 6F). Several immune pathways, such as APC co inhibition, APC co stimulation, CCR, checkpoint, cytolytic activity, human leukocyte antigen (HLA), inflammation-promoting, parainflammation, T cell co-inhibition, T cell co-stimulation, Type I IFN response, and type II IFN response, scored higher in the low-risk group than in the high-risk group (Figure 6G). Furthermore, when comparing immune checkpoint activation between high-risk and low-risk groups, we noticed that most immune checkpoints displayed a significant difference in the low-risk than the high-risk group (Figure 6H), such as *CD48*, *BTLA*, *CD40LG*, *PDCD1LG2*, *CTLA-4*, P*DCD1* (*PD-1*), *TIGIT*, and *CD70*. These findings suggest that the immune system in the low-risk group is more active and thus more responsive to immunotherapy.

### Analyses of the risk score’s relationships to the tumor Immune Dysfunction and Exclusion, m6A-related genes, and stem cell-like features

**FIGURE 7:**
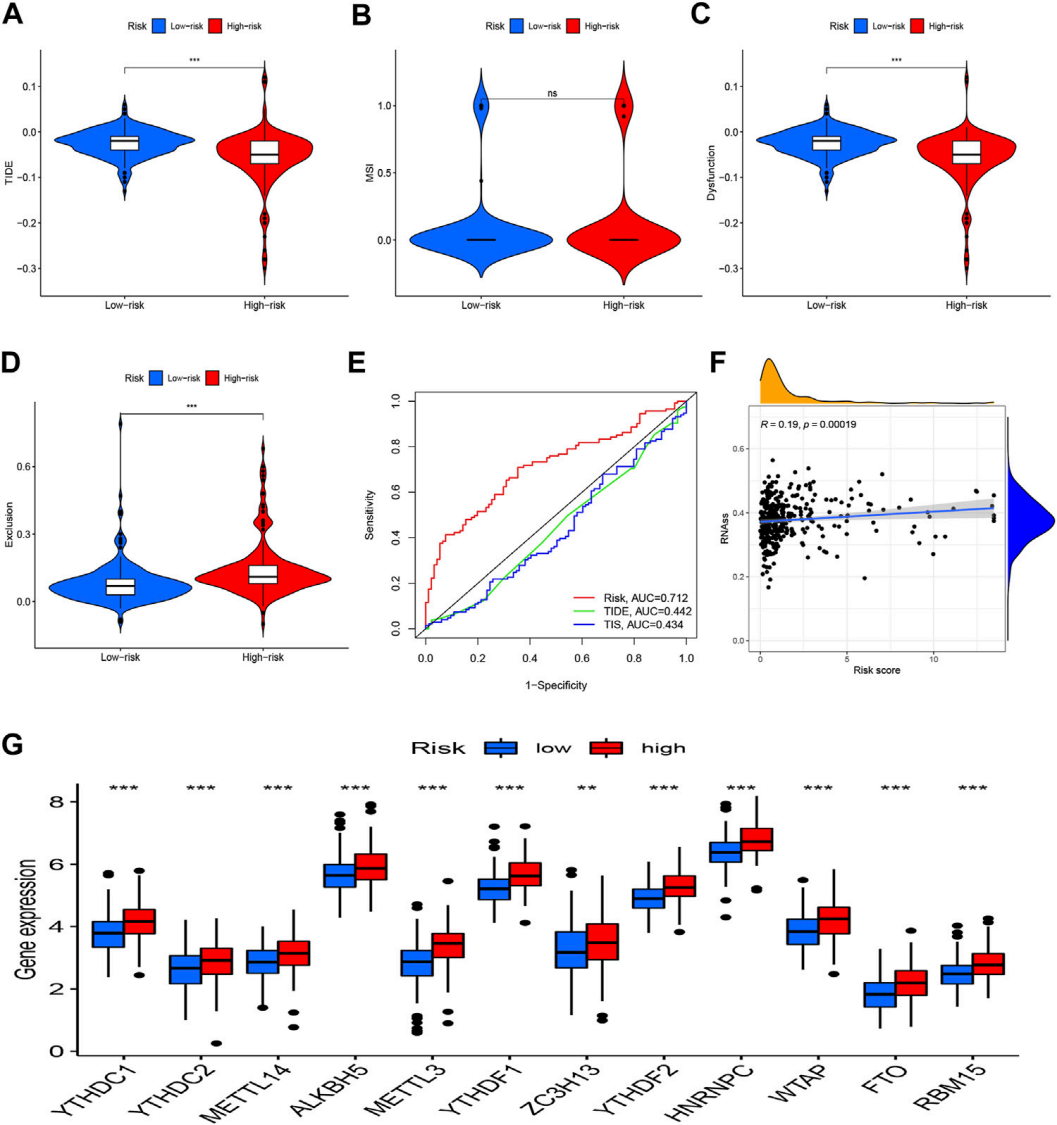
TIDE, m6A-related genes, and tumor stemness. **(A)** TIDE score between two groups; **(B)** MSI score between two groups; **(C)** Dysfunction score between two groups; **(D)** Exclusion score between two groups; **(E)** ROC analysis of risk model, TIDE and TIS; **(F)** Correlation analysis between the RNA stemness and the risk model; **(G)** Expression of m6A-related genes in risk groups. *p < 0.05, **p < 0.01, ***p < 0.001.

Moreover, *TIDE* was utilized to observe the clinical efficacy of immunotherapy in high-risk and low-risk groups. We found that the high-risk patients had a lower *TIDE* score and were more likely to benefit from immunotherapy compared to low-risk group (Figures 7A–D). Additionally, the risk model’s AUC was higher than that of TIDE and 18-gene T-cell-inflamed signature (TIS), suggesting that it has a better predictive value for HCC (Figure 7E). A Wilcoxon test was performed to investigate the association between the risk score and m6A-related genes. The boxplot demonstrates that most m6A-related genes were expressed more highly in the high-risk group (Figure 7G). Additionally, there was a strong positive correlation between the RNA stemness score and the risk model (Figure 7F).

### Cluster analysis based on prognostic NRlncRNAs

**FIGURE 8:**
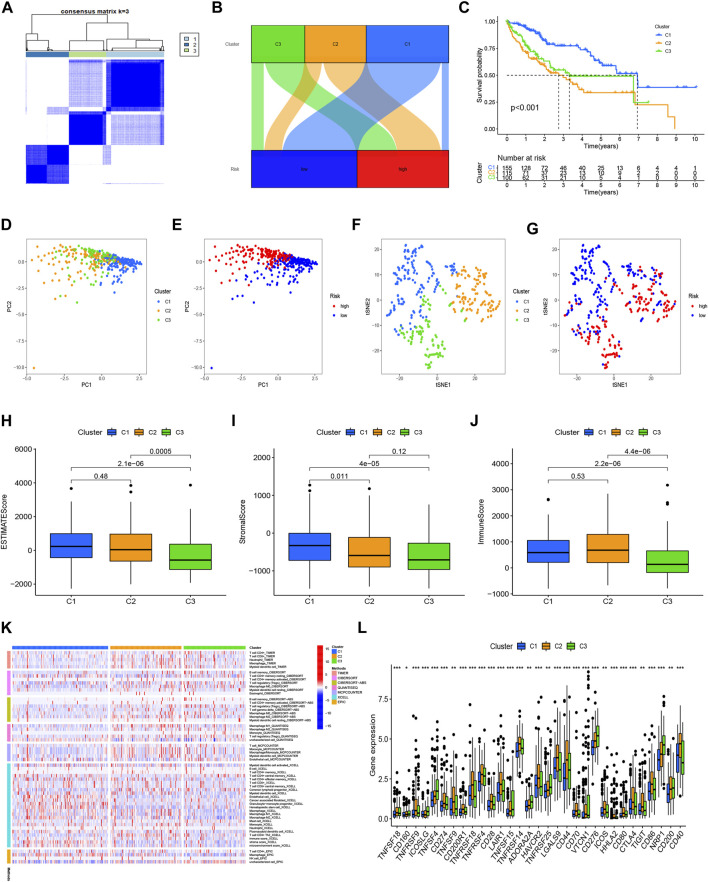
Clusters analysis. **(A)** Patients divided into three clusters; **(B)** Sankey diagram; **(C)** K-M survival curves of OS in clusters; **(D,E)** The PCA of risk groups and clusters; **(F,G)** The t-SNE of risk groups and clusters; **(H–J)** Immune-related scores in clusters; **(K)** Heatmap of immune cells in clusters; **(L)** Different expression of checkpoints in clusters.

In order to compare the immune microenvironments and responses in various tumor subtypes, cluster analysis was applied to acquire the cluster subtypes. Based on the six NRlncRNAs constituting the risk model, we ultimately classified the patients into three clusters using the “ConsensusClusterPlus” package (Figure 8A). According to the Sankey diagram (Figure 8B), the majority of the patients in the low-risk group were regrouped into cluster 1. In contrast, most of the patients in clusters 2 and 3 belonged to the high-risk group. The survival analysis revealed that cluster 1 had a better OS (*p* < 0.001) than clusters 2 and 3. (Figure 8C). According to the PCA results, we obtained the PCA graph of the risk groups and cluster groups (Figures 8D,E), and t-SNE confirmed that the three clusters could be discriminated unambiguously (Figures 8F,G). Based on the correlation among clusters, we conducted the immunological factors and the TME analysis. The boxplot revealed that cluster 1 had higher immunological, stromal, and ESTIMATE scores than clusters 2 and 3 (Figures 8H–J). The heatmap displays the variations in invading immune cells in the clusters based on examining immune infiltration by severe platforms (Figure 8K). Additionally, cluster 1 showed reduced expression of immunological checkpoints like *TNFRSF18*, *LAG3*, *CD244*, and *TNFRSF14* (Figure 8L).

### Tumor mutation burden calculation and mutation analysis

**FIGURE 9:**
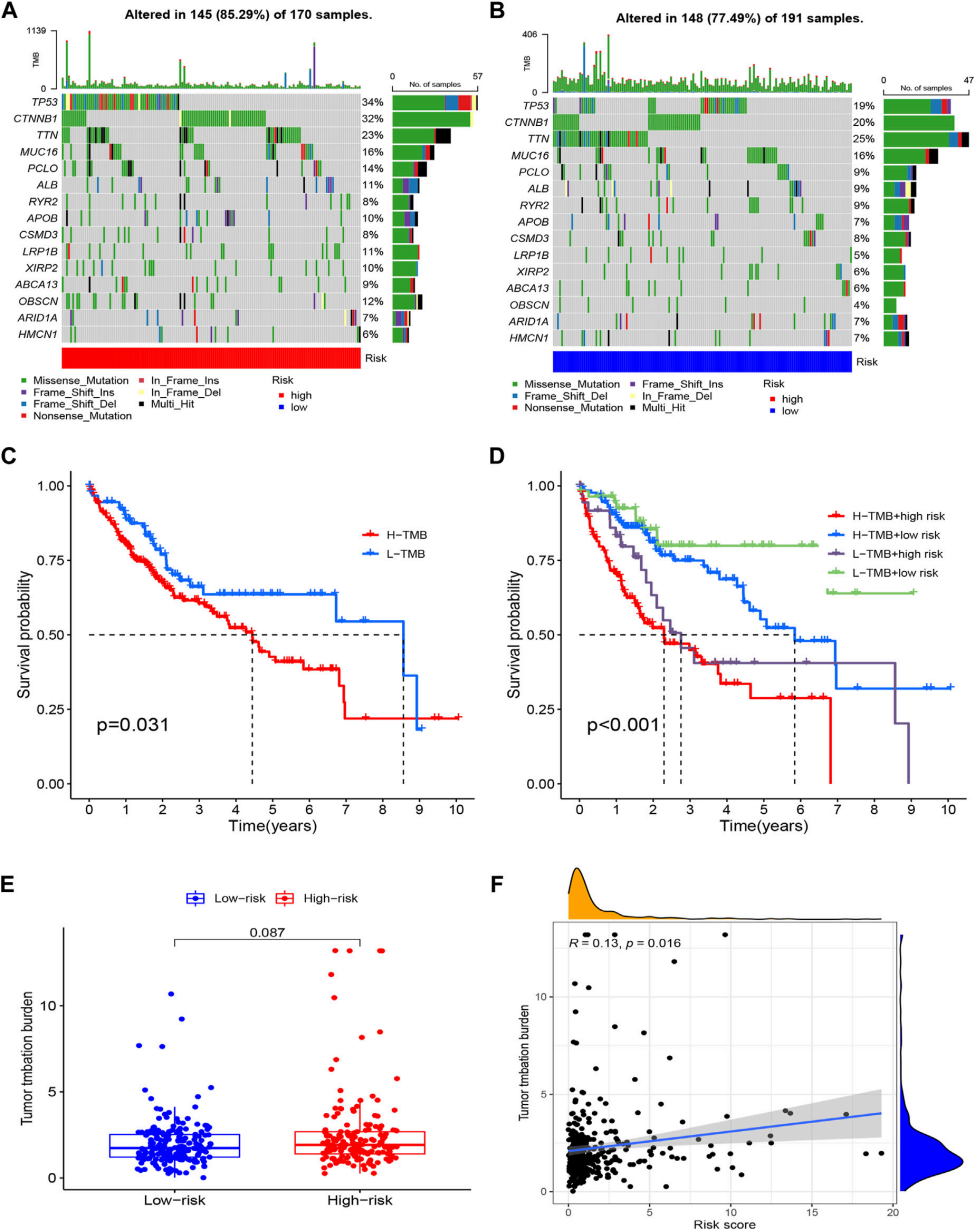
Tumor Mutation Burden Calculation Analysis in the risk groups. **(A)** Tumor somatic mutation waterfall graph in high-risk group; **(B)** Tumor somatic mutation waterfall graph in low-risk group; **(C)** Kaplan–Meier survival analyses of TMB with OS; **(D)** Kaplan–Meier survival analyses of TMB and risk group on OS; **(E)** The relationship between TMB and risk groups; **(F)** Correlation analysis of the risk score and tumor mutation load.

According to our research, 145 (85.29%) of 170 patients in the high-risk group had a wider TMB than in the low-risk group. The most genetic changes were found in *PIK3CA*, *TP53*, *TTN*, and *MUC16*, with mutation frequencies in each of these genes exceeding 15%. Results suggested that a correlation exists between somatic mutations and riskScore (Figures 9A,B). According to the survival curves, the low-TMB group possessed a better OS compared to the high-TMB group (Figure 9C). In addition, we found that the tumor mutation load (TMB) and riskScore had a strong correlation (R = 0.13, *p* = 0.016) (Figure 9F); the higher the riskScore, the higher the TMB (Figure 9E). The patients with the lowest riskScore and lowest TMB had the greatest prognosis, while those with the highest riskScore and highest TMB had the poorest prognosis when the two parameters were combined (Figure 9D).

### Prediction of chemotherapy or target agent response and analysis of quantitative real-time polymerase chain reaction

**FIGURE 10:**
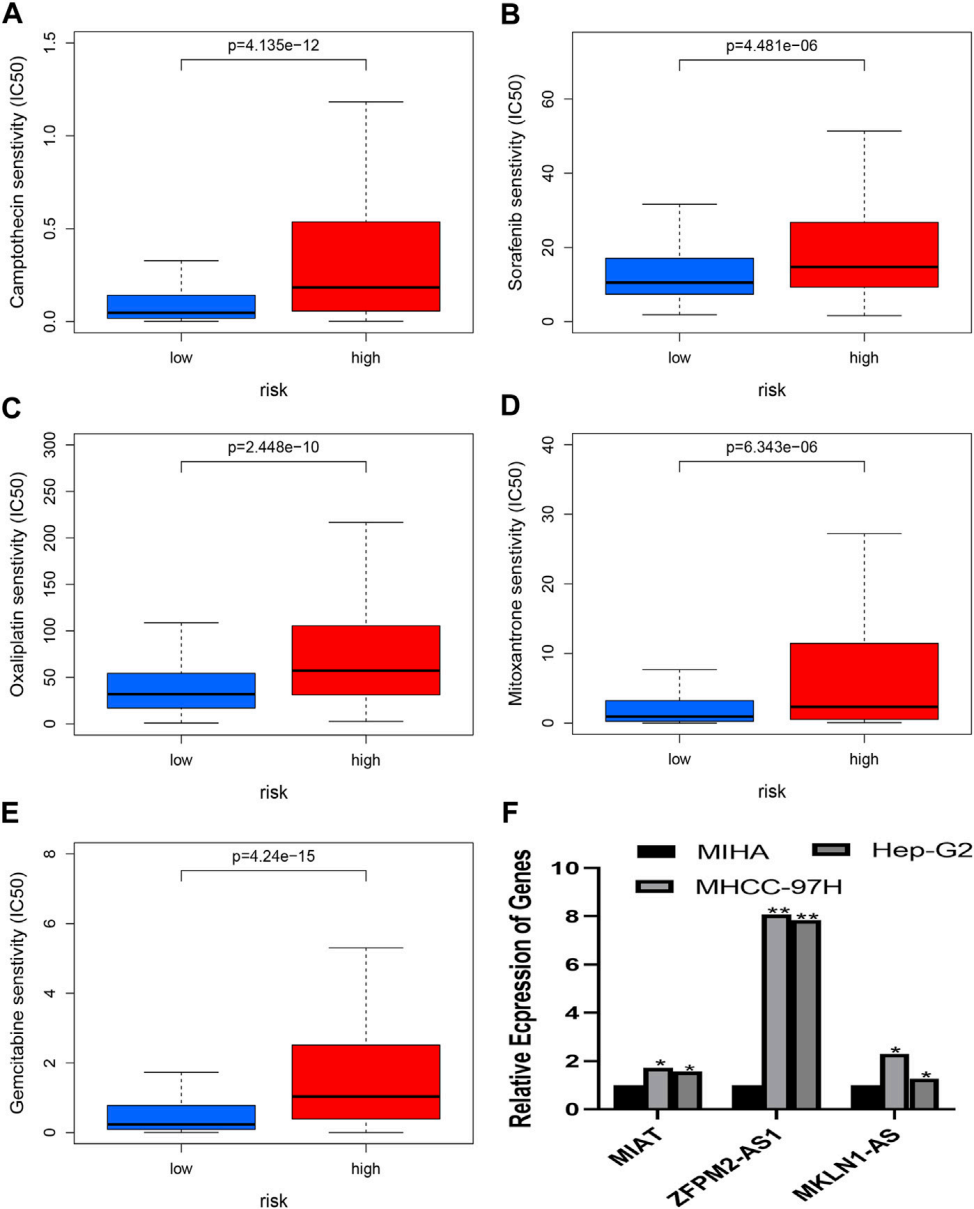
**(A–E)** presents the drug effectiveness of different risk groups. **(F)** presents the results of RT-PCR. **(A)** Camptothecin; **(B)** Sorafenib; **(C)** Oxaliplatin; **(D)** Mitoxantrone; **(E)** Gemcitabine; **(F)** Expression of *MIAT*, *ZFPM2-AS1* and *ZFPM2-AS1* in HCC cells.

The chemotherapeutic response was evaluated in HCC patients using the IC50 values of several chemotherapy drugs. In contrast to high-risk patients, low-risk patients had significantly lower IC50 values for Mitoxantrone, Gemcitabine, Oxaliplatin, Sorafenib, and Camptothecin, which suggested low risk is a sign of higher sensitivity to the medications mentioned above (Figures 10A–E). As a result, the *NRlncRNA* model may act as a potential indicator for chemotherapy. Based on the six genes of the risk model, three genes were selected to perform the RT-PCR assay. And we found that *MKLN1-AS, MIAT*, and *ZFPM2-AS1* were more highly expressed in HCC cell lines than in normal liver cells (Figure 10F).

## Discussion

Over 90% of primary liver cancer are hepatocellular carcinomas (HCC). HCC is the fifth most common cancer worldwide (Bray et al., 2018). HCC has a 5-years survival rate of 18%, which is second only to pancreatic cancer (Jemal et al., 2017). Numerous *lncRNAs* regulate the start and development of HCC(Liu et al., 2021). Previous research revealed that the *PDK1/AKT/Caspase 3* pathway is the mechanism through which *lncRNA-PDPK2P* promotes the development of hepatocellular carcinoma (Pan et al., 2019). A study discovered that a new lncRNA called uc.134 slows the growth of hepatocellular cancer by preventing *LATS1* from being ubiquitinated by *CUL4A* (Ni et al., 2017). The study verified that in hepatocellular carcinoma, *LncRNA NBR2* controls autophagy to suppress carcinogenesis (Sheng et al., 2021). The critical significance of *lncRNAs* in HCC has been highlighted in an increasing number of studies in recent years, although it is still unclear how they relate to one another. This is a hot area for further study.

Numerous cancers, including hepatocellular carcinoma, have been demonstrated to progress in significant part due to necroptosis. Cell swelling, organelle malfunction, and plasma membrane rupture are some of its morphological traits similar to necrosis (Seo et al., 2021). *RIPK3* (necroptosis-related molecules) orchestrates the breakdown of fatty acids in tumor-associated macrophages and the development of hepatocarcinogenesis (Wu et al., 2020). Necroptosis-associated compounds (*CIAPs*) are crucial in lowering macrophage programmed necrosis, which helps regulate pathogens (McComb et al., 2012). *RIPK3* activation causes cancer cells to exhibit TRIM28 derepression and improves the anti-tumor microenvironment (Park et al., 2021). Necroptosis has been linked to malignancies in a number of studies; however, the potential mechanism is still unclear. The *lncRNAs* were separated into various subgroups in our study to explore predictive markers for the first time and comprehensively investigate the relationship between the tumor microenvironment, immune cell infiltration, immunological checkpoints, and necroptosis-related *lncRNAs*. The study will guide future clinical diagnosis and therapy.

To investigate their predictive potential, this study collected 1,639 NRlncRNAs with differently expressed levels. We performed univariate, LASSO, and multivariate Cox regression analyses to build the necroptosis-related lncRNA risk model. And we acquired six *NRlncRNAs* (*MKLN1-AS, AL355574.1, AC074117.1, MIAT, ZFPM2-AS1*, and *AL365295.1*). According to ROC curves, the necroptosis-related lncRNA prognostic signature was highly accurate and reliable, and the training group’s AUC value at 1 year was 0.746. We found that Liang et al. (2021) risk model AUC value was 0.711 at 1 year, and Xing et al. risk model AUC value was 0.655 at 1 year (Deng et al., 2020). Compared to previously reported lncRNA risk models for Hepatocellular carcinoma, our lncRNA risk model is more available. The clinicopathological analysis, survival analysis, PCA, and TMB analysis suggested that this model has good sensitivity for predicting the survival of HCC patients. Results also showed that the model might be used as an independent factor for HCC patients. These results suggested a potential link between tumor immune infiltration and Necroptosis-related *lncRNAs*. As a result, our study also examined immune-related functions. The identification of possible disease compounds may be helpful for treatment. With the help of the “ConsensusClusterPlus” package, we eventually classified the patients into three clusters based on the six *NRlncRNAs* that make up the risk model. According to the survival study, Cluster 1 outlasted the other groups (*p* < 0.001). Compared to Clusters 2 and 3, Cluster 1 had much reduced immunological checkpoint expression in the majority of cases. These findings suggest consensus clustering may be associated with the immunological microenvironment and is directly related to patient prognosis. Insights into immunotherapy for HCC patients may now be possible as a result of this. These findings might serve as a valuable benchmark for upcoming immunotherapy targets.

In previous studies, Gao et al. (2020) research has demonstrated that *LncRNA MKLN1AS* worsens the progression of hepatocellular carcinoma by acting as a molecular sponge for miR6543p and encouraging the release of hepatoma-derived growth factors. According to Lu et al. (2021) study, *TCF12* recruitment and *NFAT5* activation by *LncRNA MIAT* help melanoma cells proliferate, migrate, and invade. *LncRNA ZFPM2-AS1* interacts with *UPF1* to destabilize *ZFPM2*, which promotes the advancement of lung cancer (Han et al., 2020). There is still limited research on necroptosis-related *lncRNAs* in HCC, even though more studies are linking them to the onset and development of cancer. In this study, we first constructed a risk model and conducted a consensus clustering analysis of *lncRNAs* linked to necroptosis in HCC. Secondly, this study systematically examined lncRNA prognostic markers associated with TMB, tumor microenvironment, and immune cell infiltration for the first time. This analysis may offer new insights for future research on the prognostic role of necroptosis-related *lncRNA* markers in immunotherapy. Third, we projected a few possible drugs that might be employed to treat HCC and could be beneficial for treatment in the future.

However, Our study has certain shortcomings. First, six lncRNA can not be found in other databases (including GEO, ICGC, etc.), so we do not perform the external validation analysis. Secondly, most of our study is based on bioinformatics analysis and lacks the experiment. Therefore, the study of Necroptosis-Related lncRNAs should be further explored in the future.

## Conclusion

In this article, we comprehensively evaluated the importance of Necroptosis-related *lncRNAs* in predicting survival, and we found that Necroptosis-related *lncRNAs* play a vital role in the tumor microenvironment and immune cell infiltration. And The potential regulatory mechanisms and drug prediction of Necroptosis-related lncRNAs may provide novel insights for the treatment of HCC patients. Although our study has some limitations, it may greatly contribute to the treatment of HCC and medical progression.

## Data Availability

Publicly available datasets were analyzed in this study. The names of the repository/repositories and accession number(s) can be found in the article/Supplementary Material.

## Abbreviations

AUC, Area Under Curve; CNV, copy number variation; CXCL5, chemokine (C-X-C motif) ligand 5; CXCR2, chemokine (C-X-C motif) receptor 2; GSEA, Gene Set Enrichment Analysis; HCC, Hepatocellular carcinoma; IC50, half maximal inhibitory concentration; lncRNA, Long non-coding RNA; MIAT, myocardial infarction associated transcript; MKLN1-AS, MKLN1 antisense RNA; MLKL, Mixed Lineage Kinase Domain-Like; NAFLD, Non-alcoholic fatty liver disease; NRlncRNAs, Necroptosis-related lncRNAs; OS, Overall Survival; QRT-PCR, Quantitative Real-Time PCR; RIP1, receptor-interacting Protein 1; RIP3, receptor—interacting protein 3; ROC, Receiver Operating Characteristic, PCA Principal Component Analysis; TCGA, The Cancer Genome Atlas; TMB, tumor mutation burden; ZFPM2-AS1, ZFPM2 antisense RNA 1.
