# Supplementary figures and images for: A novel necroptosis-related lncRNA signature predicts the prognosis and immune microenvironment of hepatocellular carcinoma
Source: Front Genet. 2022 Oct 4;13:985191. doi: 10.3389/fgene.2022.985191 (PMC9576851; doi:10.3389/fgene.2022.985191)

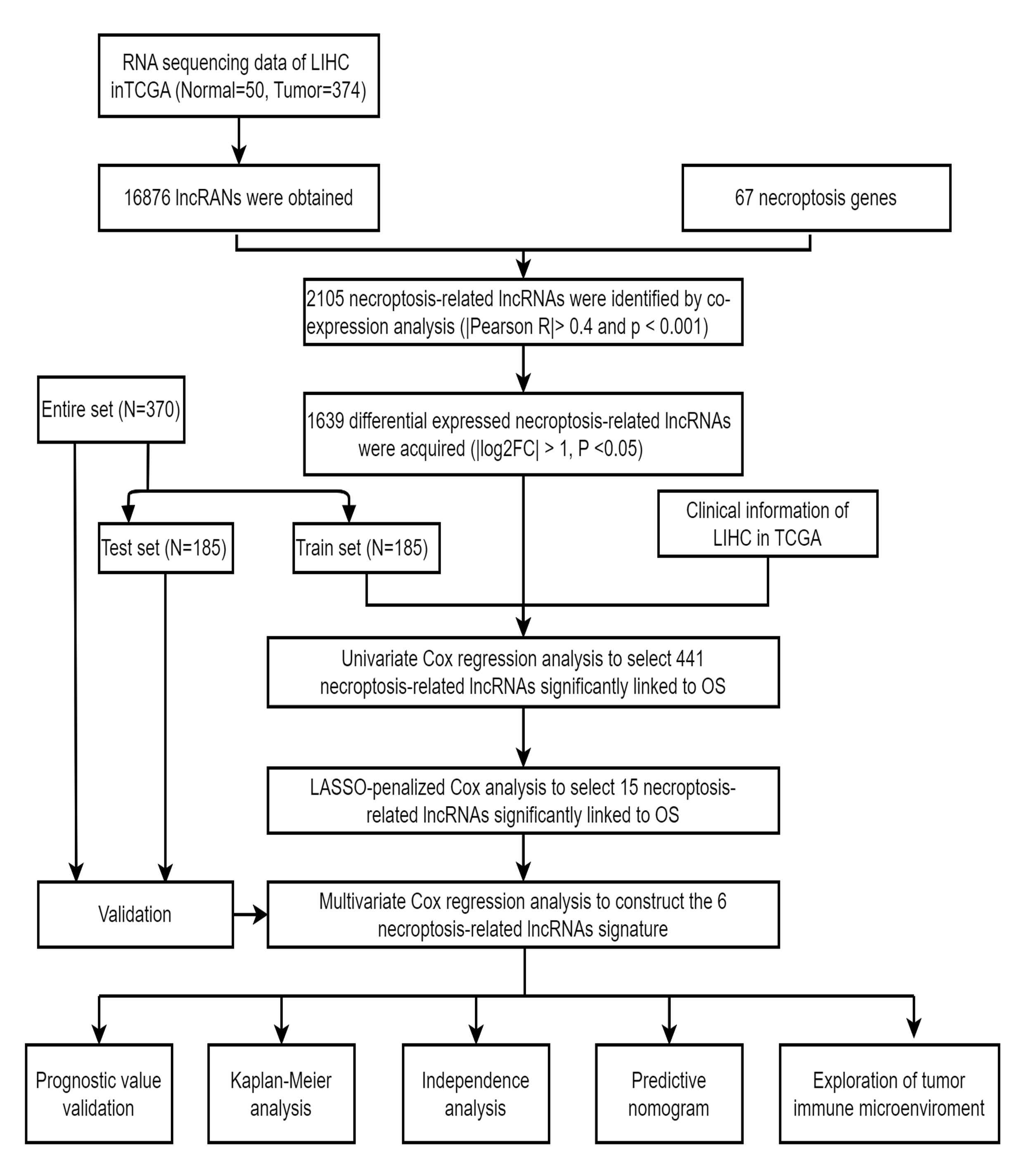

Supplement: Supplementary file 3 [file Image1.TIF]
